# Introduction of a European Central-South-Eastern West Nile Virus Lineage 2 Strain in Italy in 2023: Evidence from the First Locally Acquired Neuroinvasive Case in the Calabria Region

**DOI:** 10.3390/ijms27041809

**Published:** 2026-02-13

**Authors:** Simone Malago, Antonio Mori, Michela Deiana, Maria Vittoria Mauro, Valeria Vangeli, Giuliana Guadagnino, Silvia Accordini, Natasha Gianesini, Lorena Maria Chesini, Samuele Cheri, Sonia Greco, Francesca Greco, Jesse Julian Waggoner, Chiara Piubelli, Federico Giovanni Gobbi, Concetta Castilletti, Antonio Mastroianni

**Affiliations:** 1 PhD National Programme in One Health Approaches to Infectious Diseases and Life Science Research, Department of Public Health, Experimental and Forensic Medicine, University of Pavia, 27100 Pavia, Italy; 2 Department of Infectious, Tropical Diseases and Microbiology, IRCCS Sacro Cuore Don Calabria Hospital, 37024 Negrar di Valpolicella, Italy; 3 Microbiology Unit, “Annunziata” Hub Hospital, Azienda Ospedaliera di Cosenza, 87100 Cosenza, Italy; 4 Infectious and Tropical Diseases Unit, “Annunziata” HUB Hospital, Azienda Ospedaliera di Cosenza, 87100 Cosenza, Italy; 5 Department of Clinical and Experimental Sciences, University of Brescia, 25121 Brescia, Italy

**Keywords:** West Nile virus, WNV, lineage, sub-lineage, molecular characterization, polyprotein

## Abstract

West Nile virus lineage 2 (WNV-2) is a growing public health concern in Europe causing West Nile fever or West Nile neuroinvasive disease (WNND) with substantial morbidity and mortality; however, genomic data from southern Italy are limited despite recent expansion of autochthonous transmission. The aim of the study was to characterize the phylogenetic and molecular features of the WNV-2 strain responsible for the first autochthonous human infection reported in Calabria (2023), and two more additional WNND cases detected in 2024. Full WNV-2 genomes were generated from the three cases. Phylogenetic analysis was performed using all publicly available WNV sequences up to September 2025. Amino acid changes in the polyprotein were compared with known WNV-2 lineage and sub-lineage signatures. The three sequences formed a monophyletic group within sub-lineage WNV-2a, clustering with strains circulating in Central-South-Eastern Europe and showing closest affinity to Hungarian sequences. Non-synonymous substitutions characteristic of the Hungary 578/10 strain (NS2B-119I, NS4B-14G, NS4B-49A, and NS5-298A) were identified and were absent from Central-Northern-Western European and previously reported Italian sequences. Additional substitutions (E-159T, E-399R, and NS3-249P) corresponded to signatures from a fatal WNV-2 infection in a Great Grey Owl in Slovakia. Our study provides the first report of Central-South-Eastern European WNV-2 circulation outside Eastern Europe, supporting its likely spread through the Balkans into Italy by 2022. These findings underscore the rapid spread of WNV-2 in newly affected areas and highlight the critical need for sustained molecular surveillance.

## 1. Introduction

West Nile virus (WNV; species *Orthoflavivirus nilense*) is a mosquito-borne orthoflavivirus, mainly transmitted by Culex spp. mosquitoes. Humans are incidental dead-end hosts, and an estimated 75–80% of WNV human infections are asymptomatic, hampering accurate assessment of WNV circulation. Among symptomatic patients, most experience a self-limiting febrile illness termed West Nile fever. Approximately 1% of infections progress to West Nile neuroinvasive disease (WNND), manifesting as meningitis, encephalitis, or acute flaccid paralysis [1,2].

**Figure 1:**
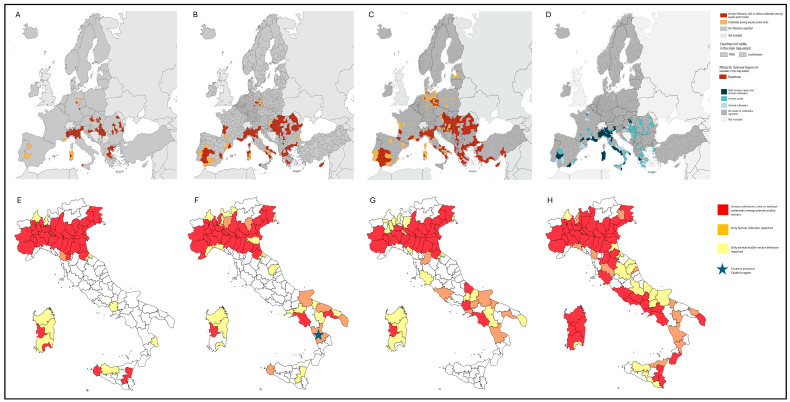
Spatial distribution of animal and human WNV infections in the EU/EEA and in Italy from 2022 to 2025. Spatial distribution in the EU/EEA and neighboring countries (**A**) 2022; (**B**) 2023; (**C**) 2024; (**D**) 2025 as of 8 October; and spatial distribution of animal, vector, and human WNV infections in Italy: (**E**) 2022; (**F**) 2023; (**G**) 2024; (**H**) 2025 as of 29 October 2025. Adapted from European Centre for Disease Prevention and Control, European Food Safety Authority (ECDC/EFSA database) [3] and from Istituto Superiore di Sanità [9]. Surveillance of West Nile virus infections in humans in Europe distribution is reported in NUTS 3 (nomenclature of territorial units for statistics, provinces level) or GAUL 1 (Global Administrative Unit Layers, subnational level 1) regions of the EU/EEA and neighboring countries. In the European maps, historical data of human infections are grouped with animal infections (Panels (**A**)–(**C**), years 2022 to 2024), while recent data (Panel (**D**), year 2025) are reported distinguishing human cases and animal cases from human and animal co-localized outbreaks.

WNV circulation in Europe continues to increase, progressively expanding into previously non-endemic areas both in northern and southern regions (Figure 1A–D, [3]) [4,5,6]. The emergence of cases in new areas highlights ongoing geographic expansion, likely driven by environmental, climatic, and ecological changes [7,8]. Italy is one of the most affected European countries, with a significant proportion of locally acquired infections, WNND cases, and fatalities. The WNND case fatality rate has remained high, at 14–20%, since 2018. In 2025, WNV was detected across 53 provinces in 14 regions, with Lazio and Campania reporting a significant increase in cases (252 and 124 cases, respectively, Figure 1E–H, [9]).

WNV is characterized by high genetic diversity, with at least nine lineages worldwide. Lineages 1 (WNV-1) and 2 (WNV-2) are the most widespread and most frequently associated with human cases [1,10]. WNV-2 currently predominates in Europe [11,12], and two main sub-lineages have been identified: WNV-2a and WNV-2b. WNV-2a, the most prevalent sub-lineage in Europe, can be further subdivided into two clusters, hereafter arbitrarily referred to as the Central-North-Western (CNW) and Central-South-Eastern (CSE) clusters. The CNW cluster likely emerged in Austria around 2006 and has been detected mainly in Germany and northern Italy, whereas the CSE cluster appears to have originated in Hungary around 2007, with a similar evolution rate, and includes strains circulating in Greece and other Central and South-Eastern European regions [6]. Continuous molecular characterization is essential to elucidate the complex viral dynamics of WNV lineages and clusters, monitor the possible introduction of more virulent/pathogenic strains, and clarify the pathogenetic mechanisms.

In September 2023, a patient presenting with severe neurological symptoms was admitted to the “Annunziata” Hub Hospital, Azienda Ospedaliera di Cosenza, in the Calabria region, the southernmost region of peninsular Italy, where laboratory testing confirmed WNV infection. This represented both the first autochthonous WNV case and the first case of WNND in Calabria. Here, we describe the molecular and phylogenetic features of the WNV strain responsible for this case as well as two additional cases detected in 2024.

## 2. Results

### 2.1. Genome Analysis of WNND Cases Detected in Calabria (2023–2024)

Blood from the WNND case diagnosed in September 2023 tested positive for WNV RNA, with a viral load of 11,155 copies/mL. The two WNND cases diagnosed at the same hospital in August and September 2024 had urine samples, with viral loads of 842,845 copies/mL and >50,000,000 copies/mL, respectively. Viral genomes were then sequenced, and for the lower-viral-load blood sample, two complementary approaches were used based on hybrid capture and amplicon-based sequencing.

### 2.2. Consensus Generation

For the 2023 case, reads generated from both sequencing methods were merged and aligned against the sequence of a WNV-2 strain isolated in the Campania region in November 2024 (GenBank ID: PQ654050). This produced a breadth of coverage of 88% at 5X and 77% at 10X. Sequencing reads from the two 2024 cases showed >99% genome coverage at 10X when aligned to the same reference. The resulting consensus sequences (WNVIRCCS-SCDC_01/2025, WNVIRCCS-SCDC_02/2025, and WNVIRCCS-SCDC_03/2025, respectively) were used for phylogenetic analysis. Complete sequencing metrics are reported in Table S1. Link to the repository of obtained sequences information is reported in Table S2.

### 2.3. Phylogenetic Analysis

**Figure 2:**
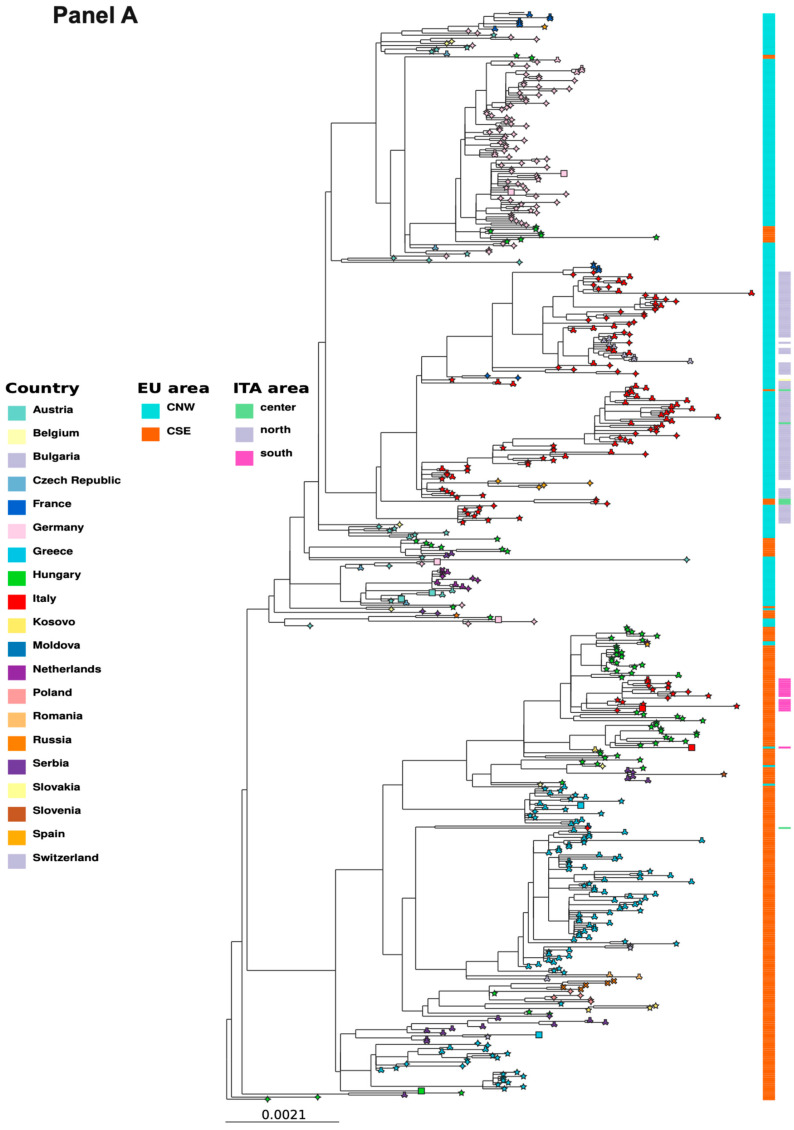

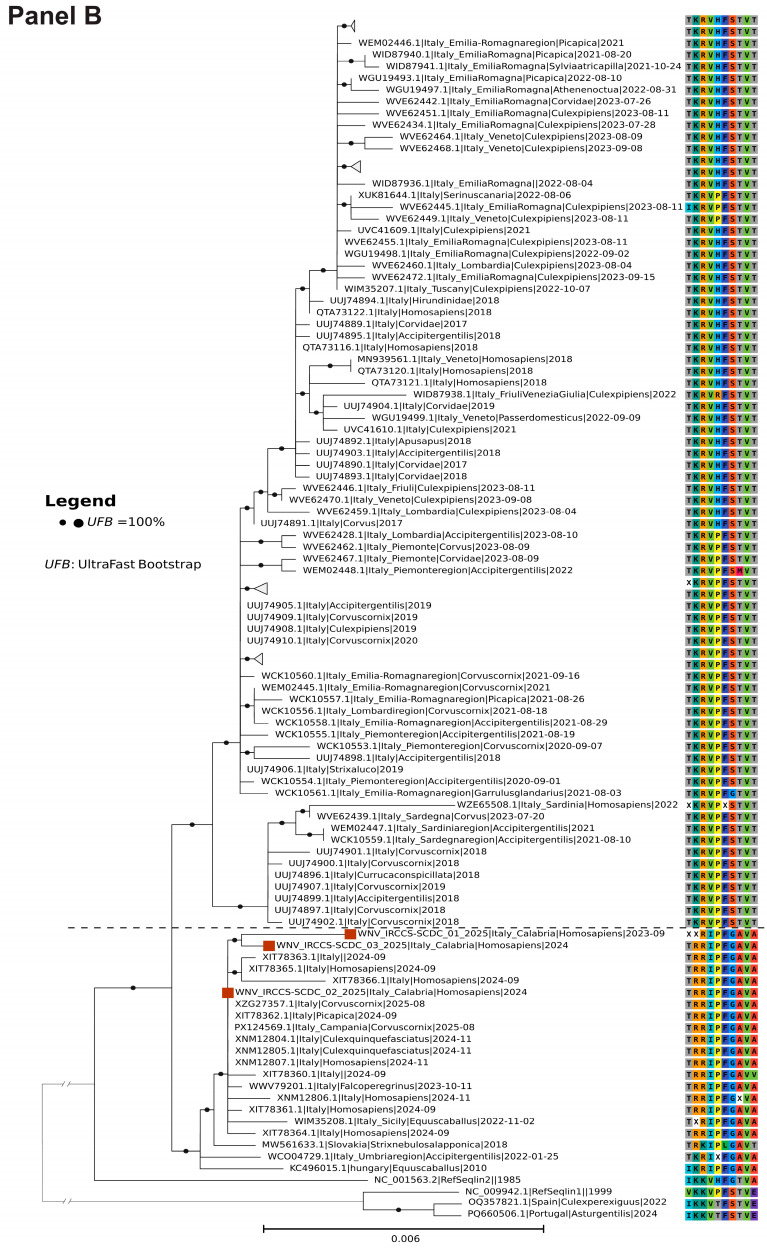
Phylogenetic analysis results. Panel (**A**): Maximum likelihood phylogenetic tree of the European WNV-2 strains. Leaf shapes represent the type of host. Colors of the leaves represent the country of collection. Geographical areas refer to the columns on the right, describing European and Italian sub-areas of WNV-2 distribution. Shapes represent the host type: bird (rhombus); human (star); mammal (square); and mosquito (inverted-wye). Outgroups and other sequences are excluded for visualization purpose. This sub-tree is part of an African–European tree (Supplementary Figure S1). Panel (**B**): Phylogenetic tree of viral strains circulating in Italy, based on complete WNV lineage 2 polyprotein sequences. Three WNV lineage 1 sequences were included as an outgroup. The Hungarian KC496015.1 and Lineage 2 reference sequences were added for comparison. On the right, amino acid residues at positions of interest for each sequence are displayed. More specifically, from left to right are positions (genes): 449 and 689 (E); 835 (NS1); 1493 (NS2B); 1754 and 1991 (NS3); 2287, 2322, and 2386 (NS4B); and 2827 (NS5) of the polyprotein. The IUPAC official one-letter code has been used for amonoacid representation (http://publications.iupac.org/pac/1984/pdf/5605x0595.pdf (accessed on 29 December 2025)). The “x” symbol indicate that the amoniacid in that position cannot be inferred by the nuceotide sequence. Sequence labels include strain identifiers, country of collection, host, and date of sampling. Dotted line indicates the separation between the south-Italian and the north-italian strains. Dots on tree branches represent bootstrap values, as per the legend. Empty triangles indicated collapsed branches. Our three sequences are indicated with brown squares on respective leaves.

Phylogenetic analysis showed that all three genomes belonged to the WNV-2, sub-lineage 2a, clustering within sequences from the European Central-South-Eastern cluster (Figure 2, Panel A). Neighboring branches included sequences from Greece, Hungary, Serbia, Russia, Romania, Slovakia, Poland, and Kosovo [6], with the most closely related genomes originating mainly from Hungary.

**Figure 3:**
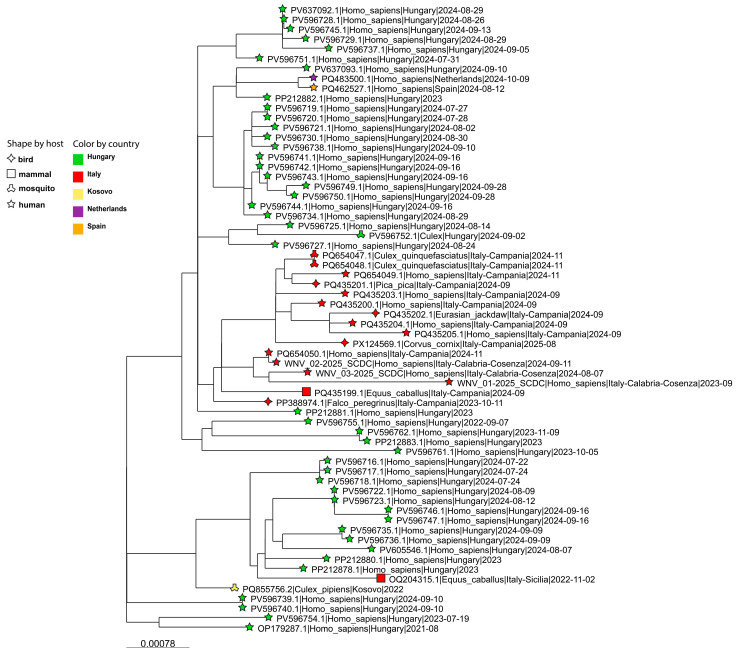
Phylogenetic sub-tree of the most closely related sequence to the 2023 and 2024 Calabria region’s cases. Phylogenetic analysis can also be directly visualized at https://microreact.org/project/wnv2-first-calabria-wnnd (accessed on 29 December 2025).

Only two of the nearest sequences were associated with countries other than Hungary, both linked to Spain. Among the Italian genomes, the closest sequences were from southern Italy, mainly from the Campania region in 2022–2025 (Figure 3). By contrast, all sequences from northern and central Italy clustered on a separate branch together with those from Sardinia, while adjacent branches were mainly composed of strains from Central-North-Western Europe, with additional genomes from other Eastern European countries.

### 2.4. Variant Analysis

Variant calling and annotation were performed using the WNV lineage 2 reference genome (GenBank ID: NC_001563.2). A total of 99 Single Nucleotide Variants (SNVs) were identified in WNVIRCCS-SCDC_01/2025, with 89 annotated as synonymous and 9 as non-synonymous substitutions. Regarding the 2024 sequences, 197 SNVs were detected in WNVIRCCS-SCDC_02/2025 (171 synonymous, 19 non-synonymous), and 138 SNVs were identified in WNVIRCCS-SCDC_03/2025 (116 synonymous, 15 non-synonymous). Among the non-synonymous variants, none were predicted as high impact by Snp-Eff. The three WNV-2 genomes detected in the Calabria region were characterized by a set of specific amino acid residues associated with south-Italian strains, clearly showing a consistent phylogenetic separation from the north-Italian strains (E-399R, NS2B-119I, NS4B-14G, NS4B-49A, and NS5-298A, Figure 2, Panel B). Furthermore, E-159T and NS3-249P, two residues of particular interest, were shared by all south-Italian cases and by a certain percentage of north-Italian ones (Table 1).

## 3. Discussion

This study investigated the phylogeny and molecular characteristics of WNV strains responsible for the first autochthonous infections reported from the Calabria region, which resulted in WNND. Phylogenetic analysis showed that the three WNV-2 genomes belong to a monophyletic clade within sub-lineage 2a that derived from a single introduction into Europe of the prototype Hungarian strain (Hungary 2004, GenBank Accession ID: DQ116961). It should be explicitly acknowledged that genuine transmission chains cannot be reliably reconstructed based solely on genomes derived from human cases. These three WNV-2 genomes clustered with CSE strains, and the most closely related Italian sequences were detected in bird, mammal, and mosquito vectors from southern Italy, mainly Campania, between 2022 and 2025 (see Figure 2, Panel B). This observation supports the hypothesis of an ongoing enzootic circulation in the macro-area, which is consistent with the human cases reported in Calabria.

Our report provides the first evidence of the circulation of a Hungary 578/10 WNV-2-like strain outside of Eastern Europe. This strain appears to have spread across Central-South-Eastern Europe over the past five years, reaching Italy through the Balkans, likely in 2022, leading to the emergence of a WNV-2 strain with epidemiologically relevant substitutions. In the Calabrian cases, we identified non-synonymous substitutions leading to amino acid signatures characteristic of the Hungary 578/10 strain, originally isolated in 2010. In particular, amino acid substitutions NS2B-119I, NS4B-14G, NS4B-49A, and NS5-298A were not found in European CNW sequences (including those from north-central Italy, Figure 2, Panel B). Additionally, E-159T, E-399R, and NS3-249P were identified as signatures of the southern-Italian cases, in common with a case of WNV neuroinvasive case in a Great Grey Owl (Slovakia 2018, GenBank Accession ID: MW561633.1) [13].

The proline at position NS3-249 has been previously associated with increased neurovirulence [14,15]. In comparison to CSE strains, approximately half of the sequences from northern Italy carry a valine at this position (Figure 2, Panel B). NS3 encodes the helicase protein, and a proline at position 249 enhances mortality in birds and increases viremia, facilitating virus transmission [14]. In silico structural modeling suggests that NS3-249P, NS3-249A, and NS3-249T mutants have similar kinetic profiles at 28 °C and 37 °C, but only NS3-249P uniquely retains activity at 42 °C (avian body temperature), likely contributing to greater protein stability and viral transmissibility [14,15]. Despite its association with higher bird mortality, NS3-249P appears to be under positive selective pressure; it has been independently introduced into WNV-1 and -2 strains and has preceded human outbreaks in geographically distant countries over the past 70 years [14,16]. Furthermore, in vitro and in vivo studies in mice showed a similar neurovirulence for the Hungarian WNV-578/10 strain and representative strains of WNV-2 European CNW cluster strains [17], although this similarity is not reflected in the observed differences in human lethality rate between the CSE cluster (higher) versus the CNW cluster (lower) [18]. Substitution E-159T in the envelope protein is considered a determining factor of WNV neurovirulence due to its impact on the host response and neuronal cell degeneration [19,20]. This substitution, absent in the Hungarian 578/10 strain (Figure 2, Panel B) but present in Southern Italian strains, had previously been identified in several Italian WNV-2 genomes isolated between 2011 and 2014 in northern Italy [14,15]. Furthermore, the combination of E-159T and E-399R was observed not only in the sequences from the Calabria region, but also in the WNV-2 strain isolated in Slovakia in 2018 from a captive Great Grey Owl, associated with a fatal infection [13]. While experimental and epidemiological evidence suggests a possible role of these substitutions in influencing host response and neuronal cell damage, definitive conclusions on their functional impact cannot be drawn.

## 4. Materials and Methods

This study included the full genome characterization and phylogenetic and variant analysis of WNV derived from three WNND human cases diagnosed in the Calabria Region (South Italy) in 2023–2024.

### 4.1. Clinical Samples and Laboratory Diagnosis

Laboratory diagnosis of WNND was performed at the “Annunziata” Hub Hospital in Cosenza, Italy. For the first autochthonous case identified in Calabria, collected on September 2023, cerebrospinal fluid (CSF) and blood samples were analyzed using the one-step real-time RT-PCR WNV ELITe MGB^®^ Kit (ELITechGroup SAS, Puteaux, France), following the manufacturer’s instructions. Both samples tested positive for WNV RNA, with viral loads of <500 copies/mL in CSF and 11,155 copies/mL in blood. In addition, two additional WNND cases diagnosed in 2024 at the same hospital were also included. For both patients, urine samples were collected in August and September 2024, respectively, with viral loads of 842,845 copies/mL and >50,000,000 copies/mL.

### 4.2. Viral Full Genome Sequencing Analysis

Sample pre-processing, sequencing, and bioinformatic analyses were carried out at IRCCS Sacro Cuore Don Calabria Hospital (Negrar di Valpolicella, Verona, Italy). Analyses were performed on the same samples used for the laboratory diagnosis.

Both whole-blood and urine samples were diluted 1:4 in PBS to improve extraction efficiency and nucleic acids were extracted using the EZ1^®^ DSP Virus kit on the Qiagen EZ1 Advanced XL, (Qiagen, Hilden, Germany). RNA quantity and quality were assessed using the Qubit RNA HS assay kit (Invitrogen, Thermo Fisher Scientific, Inc., Waltham, MA, USA) and the High Sensitivity RNA ScreenTape on the 4200 TapeStation System (Agilent Technologies Inc., Santa Clara, CA, USA).

Due to the low viral load of the 2023 sample, two sequencing approaches were employed: (i) Illumina RNA Prep kit with enrichment via the Illumina Viral Surveillance Panel (VSP) hybridization capture probes (Illumina, CA, USA); and (ii) a 400 bp tiled-amplicons panel, as described by Diagne and colleagues [21]. Amplicons were then processed using Illumina DNA Prep kit. Libraries were loaded onto an Illumina P1 flow cell and sequenced in 2 × 150 mode, on a NextSeq1000 instrument (Illumina).

Reads from both runs were merged and processed via kraken2 v2.1.3 (https://github.com/DerrickWood/kraken2, access 1 September 2025) to eliminate human reads, then trimmed via fastp v0.23.4 (https://github.com/OpenGene/fastp#fastp, access 1 September 2025) and aligned via bwa-mem2 v2.2.1 (https://github.com/bwa-mem2/bwa-mem2, access 1 September 2025) against the GenBank (NCBI, https://www.ncbi.nlm.nih.gov/genbank/, access 1 September 2025) reference sequence NC_001563.2. This alignment yielded a breadth of coverage of 80% at 5X and 70% at 10X. Consensus sequence was retrieved using iVar consensus v1.4.4 (https://github.com/andersen-lab/ivar, access 3 September 2025) with minimum base quality set to 10 (-q 10), minimum percentage of base frequency to 0.7 (-t 0.7), and minimum depth of coverage to 5 reads (-m 5). Alignment statistics were evaluated through samtools “coverage” and “flagstat” v1.22. Inspection of the alignment with Integrative Genomics Viewer (IGV) v2.16.2 (https://github.com/igvteam/igv, access 3 September 202525) revealed a high number of mismatches, prompting realignment against a more closely related sequence (GenBank accession ID: PQ654050), corresponding to a WNV-2 strain detected in Campania (Italy) in November 2024 (see phylogenetic analyses paragraph). This second alignment produced improved coverage (88% at 5X and 77% at 10X); the corresponding consensus sequence was therefore used for phylogenetic analyses.

For the two 2024 samples, which showed high viral loads, only the Illumina hybrid capture protocol was applied. Consensus sequences were generated following the bioinformatic workflow applied to the 2023 sample.

### 4.3. Phylogenetic Analysis

All available European and African WNV-2 whole-genome sequences were downloaded from the NCBI Virus database (https://www.ncbi.nlm.nih.gov/labs/virus/vssi/#/, last accessed on 5 September 2025; sequence length ≥ 10.5 Kb; nucleotide completeness: complete; ambiguous characters ≤ 10). The dataset included our newly generated consensus genomes, the downloaded sequences, and the WNV-2 reference genome (NC_001563.2). Phylogenetic analysis included WNV sequences deposited in the NCBI Virus database from the earliest European and African sequences through to September 2025. Most of these were from Italy (*n* = 124), Greece (*n* = 112), Germany (*n* = 99), Russia (*n* = 87), and Hungary (*n* = 76), and were predominantly associated with mosquito (~36%), human (~29%), and bird (~27%) hosts. Multiple sequence alignment was performed using mafft v7.505 (https://github.com/GSLBiotech/mafft, last accessed on 10 September 2025). The resulting multiple sequence alignment (MSA) was used to infer a phylogenetic tree with iq-tree v2.3.5 (https://github.com/iqtree/iqtree2, last accessed on 22 September 2025) using WNV lineage 1 sequences as outgroup and applying 1000 rounds of ultrafast bootstrap (-B 1000), 1000 rounds of SH-aLRT test (-alrt 1000), and testing the selection for the best-fit substitution model (-m MFP) The best-fit model was: GTR + F + I + R4. Based on the resulting tree, the genome showing the closest genetic similarity to our sequences was the “PQ654050” (GenBank accession ID). This sequence was therefore used for the final alignment and consensus generation, following the same analytical pipeline described above. Trees were visualized via microreact (https://microreact.org/; https://microreact.org/project/wnv2-first-calabria-wnnd, last accessed 12 February 2026).

### 4.4. Variant Analysis

Variant calling was performed via bcftools v 1.18-9 (https://github.com/samtools/bcftools, last accessed on 10 September 2025) and variants were annotated with SnpEff v5.2c (https://pcingola.github.io/SnpEff/, last accessed on 15 September 2025) using the reference genome NC_001563.2 for annotation purpose. Moreover, amino acid residue comparison was carried out using the Hungarian 578/10 strain (GenBank accession ID: KC496015) as a reference and representative of the CSE European WNV-2 cluster.

To carry out this analysis, all available Italian WNV lineage 2 complete polyprotein sequences (length aa > 3400) were downloaded from the NCBI Virus database (last accessed on 05 September 2025) and combined with the three sequences generated in our center. Additional sequences of epidemiological interest were included: the isolate from a Great Grey Owl from Slovakia (GenBank accession ID: MW561633) [15], the WNV lineage 2 reference (GenBank accession ID: NC_001563.2), and three WNV lineage 1 sequences used as outgroup. In total, 134 amino acid sequences were included and aligned via mafft v7.505. Tree was visualized via TreeViewer v2.2.0 (https://github.com/arklumpus/TreeViewer, last accessed on 29 December 2025) [22].

## 5. Conclusions

In recent years, southern Italy has experienced an abrupt increase in locally acquired WNV-2 WNND cases, leading to the major epidemic observed in the Lazio and Campania regions in 2025 (Figure 1) [23,24,25,26,27]. Although preliminary data do not allow us to determine whether the detected Calabria strains exhibit enhanced neurovirulence, further investigations are warranted to clarify this point. Overall, our findings contribute to a better understanding of WNV-2 transmission dynamics in Europe and highlight the need for sustained molecular surveillance to monitor viral evolution and diversity.

## Figures and Tables

**Table 1:** Positions of investigated residues of interest in WNV-2 polyprotein. Rows report amino acid residues of the following sequences: the Central-South-Eastern clade ancestor from Hungary (GenBank Accession ID: KC496015.1), a Great Grey Owl case of WNND (GenBank Accession ID: MW566133.1) from Slovakia, a representative set of the south-Italian cases (*n* = 19), and finally, a representative set of the central-northern-Italian WNV-2 strains (*n* = 109).

Gene Name	E		NS1	NS2B	NS3		NS4B			NS5
Position in Polyprotein	159	399	44	119	249	486	14	49	113	298
**KC496015.1 (Hungary 578/10)**	I	K	R	I	P	F	G	A	V	A
**MW561633.1 (Great Grey Owl, Slovakia 2018)**	T	R	K	I	P	L	G	A	V	T
**South Italy (*n* = 19)**	T	R	R	I	P	F	G	A	V	A
**North-Central Italy (*n* = 109)**	T	K	R	V	P/H	F	S	T	V	T

## Data Availability

Sequence raw reads were uploaded in the European Nucleotide Archive (ENA, EMBL-EBI) (project code PRJEB104938). Consensus sequences for WNVIRCCS-SCDC_01/2025, WNVIRCCS-SCDC_02/2025, and WNVIRCCS-SCDC_03/2025 were uploaded in GenBank (NCBI, accession IDs: PX289511, OZ374057.1, and OZ374058.1, respectively).

## Supplementary Materials

The following supporting information can be downloaded at: https://www.mdpi.com/article/10.3390/ijms27041809/s1.

## References

[1] Gould C.V., Staples J.E., Guagliardo S.A.J., Martin S.W., Lyons S., Hills S.L., Nett R.J., Petersen L.R. (2025). West Nile Virus: A Review. JAMA.

[2] Patel H., Sander B., Nelder M.P. (2015). Long-term sequelae of West Nile virus-related illness: A systematic review. Lancet Infect. Dis..

[3] European Centre for Disease Prevention and Control West Nile Virus Infection. https://www.ecdc.europa.eu/en/west-nile-virus-infection.

[4] European Centre for Disease Prevention and Control (ECDC), European Food Safety Authority (EFSA) (2025). Surveillance of West Nile virus infections in humans and animals in Europe, monthly report – data submitted up to 3 September 2025. EFSA J..

[5] Mencattelli G., Ndione M.H.D., Silverj A., Diagne M.M., Curini V., Teodori L., Di Domenico M., Mbaye R., Leone A., Marcacci M. (2023). Spatial and temporal dynamics of West Nile virus between Africa and Europe. Nat. Commun..

[6] Lu L., Zhang F., Oude Munnink B.B., Munger E., Sikkema R.S., Pappa S., Tsioka K., Sinigaglia A., Dal Molin E., Shih B.B. (2024). West Nile virus spread in Europe: Phylogeographic pattern analysis and key drivers. PLoS Pathog..

[7] Erazo D., Grant L., Ghisbain G., Marini G., Colón-González F.J., Wint W., Rizzoli A., Van Bortel W., Vogels C.B.F., Grubaugh N.D. (2024). Contribution of climate change to the spatial expansion of West Nile virus in Europe. Nat. Commun..

[8] European Centre for Disease Prevention and Control (2024). Surveillance of West Nile Virus Infections in Humans and Animals in Europe, Monthly Report. https://wnv-monthly.ecdc.europa.eu/archive/wnv-2024.html.

[9] Istituto Superiore di Sanità Sorveglianza Integrata Del West Nile e Usutu Virus. Bollettino N.16 del 30 Ottobre 2025. https://www.epicentro.iss.it/westNile/bollettino.

[10] Fall G., Di Paola N., Faye M., Dia M., Freire C.C.D.M., Loucoubar C., Zanotto P.M.D.A., Faye O., Sall A.A. (2017). Biological and phylogenetic characteristics of West African lineages of West Nile virus. PLoS Negl. Trop. Dis..

[11] Barzon L., Montarsi F., Quaranta E., Monne I., Pacenti M., Michelutti A., Toniolo F., Danesi P., Marchetti G., Gobbo F. (2022). Early start of seasonal transmission and co-circulation of West Nile virus lineage 2 and a newly introduced lineage 1 strain, northern Italy, June 2022. Eurosurveillance.

[12] García San Miguel Rodríguez-Alarcón L., Fernández-Martínez B., Sierra Moros M.J., Vázquez A., Julián Pachés P., García Villacieros E., Gómez Martín M.B., Figuerola Borras J., Lorusso N., Ramos Aceitero J.M. (2021). Unprecedented increase of West Nile virus neuroinvasive disease, Spain, summer 2020. Eurosurveillance.

[13] Peňazziová K., Korytár L., Pastorek P., Pistl J., Rusňáková D., Szemes T., Čabanová V., Ličková M., Boršová K., Klempa B. (2021). Genetic characterization of a neurovirulent west nile virus variant associated with a fatal great grey owl infection. Viruses.

[14] Brault A.C., Huang C.Y.-H., Langevin S.A., Kinney R.M., Bowen R.A., Ramey W.N., Panella N.A., Holmes E.C., Powers A.M., Miller B.R. (2007). A Single Positively Selected West Nile Viral Mutation Confers Increased Virogenesis in American Crows. Nat. Genet..

[15] Barzon L., Papa A., Lavezzo E., Franchin E., Pacenti M., Sinigaglia A., Masi G., Trevisan M., Squarzon L., Toppo S. (2015). Phylogenetic characterization of Central/Southern European lineage 2 West Nile virus: Analysis of human outbreaks in Italy and Greece, 2013–2014. Clin. Microbiol. Infect..

[16] Langevin S.A., Bowen R.A., Reisen W.K., Andrade C.C., Ramey W.N., Maharaj P.D., Anishchenko M., Kenney J.L., Duggal N.K., Romo H. (2014). Host competence and helicase activity differences exhibited by west Nile viral variants expressing NS3-249 amino acid polymorphisms. PLoS ONE.

[17] Visser I., Marshall E.M., Agliani G., Rissmann M., van den Brand J.M., Koopmans M.P., Rockx B. (2024). In Vitro and in vivo characterization of a novel West Nile virus lineage 2 strain. Npj Viruses.

[18] Vlaskamp D.R., Thijsen S.F., Reimerink J., Hilkens P., Bouvy W.H., Bantjes S.E., Vlaminckx B.J., Zaaijer H., van den Kerkhof H.H., Raven S.F. (2020). First autochthonous human West Nile virus infections in the Netherlands, July to August 2020. Eurosurveillance.

[19] McMullen A.R., Albayrak H., May F.J., Davis C.T., Beasley D.W.C., Barrett A.D.T. (2013). Molecular evolution of lineage 2 West Nile virus. J. Gen. Virol..

[20] Kobayashi S., Kaneko C., Kawakami R., Hasebe R., Sawa H., Yoshii K., Kariwa H. (2020). Amino acid 159 of the envelope protein affects viral replication and T-cell infiltration by West Nile virus in intracranial infection. Sci. Rep..

[21] Diagne M.M., Ndione M.H.D., Mencattelli G., Diallo A., Ndiaye E.H., Di Domenico M., Diallo D., Kane M., Curini V., Top N.M. (2023). Novel Amplicon-Based Sequencing Approach to West Nile Virus. Viruses.

[22] Bianchini G., Sánchez-Baracaldo P. (2024). TreeViewer: Flexible, modular software to visualise and manipulate phylogenetic trees. Ecol. Evol..

[23] Loconsole D., Centrone F., Sallustio A., Casulli D., Colella V., Mongelli O., Venturi G., Bella A., Marino L., Martinelli D. (2023). Abrupt Increase in Detection of Locally Acquired West-Nile-Virus-Lineage-2-Mediated Neuroinvasive Disease in a Previously Non-Endemic Area of Southern Italy (2023). Viruses.

[24] Esposito N., Viceconte G., Festa L., Alfè F.A., Carriero C., Codella A.V., Glielmo A., Forgione L., Buonomo A.R., Muccio F.C. (2025). Outbreak of Human Neuroinvasive West Nile Virus Infection in Campania, Italy, August–September 2024. Vector-Borne Zoonotic Dis..

[25] Mussetto I., Bongiovanni A., Colavita F., Giambi C., Sala M.G., Del Borgo C., Carletti F., Scicluna M.T., Zerbetto A., Corpolongo A. (2025). Outbreak of autochthonous West Nile virus infection in Lazio region, Italy, July to August 2025: Preliminary investigation. Eurosurveillance.

[26] Gucciardi F., De Grazia S., Guercio A., La Russa F., Di Pasquale M.L., Bonura F., Monaco F., Spedicato M., Bonfini B., Savini G. (2025). Introduction of West Nile virus lineage 2 leads to neuroinvasive cases in humans and horses in Sicily, 2022–2023. Vet. Res..

[27] Italian Ministry of Health Piano Nazionale di Prevenzione, Sorveglianza e Risposta Alle Arbovirosi (PNA) 2020–2025. https://www.salute.gov.it/portale/documentazione/p6_2_2_1.jsp?id=2947.

