# Supplementary material for: Introduction of a European Central-South-Eastern West Nile Virus Lineage 2 Strain in Italy in 2023: Evidence from the First Locally Acquired Neuroinvasive Case in the Calabria Region
Source: Int J Mol Sci. 2026 Feb 13;27(4):1809. doi: 10.3390/ijms27041809 (PMC12941303; doi:10.3390/ijms27041809)
Supplement: Supplementary file 1 [file ijms-27-01809-s001.zip › ijms-4101198-supplementary.pdf]

SUPPLEMENTARY MATERIAL

Supporting information alongside the article [**Introduction of a European Central-South-Eastern West Nile Virus Lineage 2 strain in Italy in 2023: evidence from the first locally acquired neuroinvasive case in the Calabria region.**] by Simone Malagò et al., on behalf of the authors, who remain responsible for the accuracy and appropriateness of the content.

Sequencing quality

As shown in Table S1, sample WNV$_{\text{IRCCS-SCDC_01/2025}}$ produced approximately 6.3 million reads via the hybrid capture sequencing method (Illumina VSP panel), in 2x150 configuration. The short amplicon panel generated approximately 19 million reads, with amplicons of approximately 400bp in length. Sample WNV$_{\text{IRCCS-SCDC_02/2025}}$ produced a total of 4,626,870 reads via the hybrid capture sequencing method (Illumina VSP panel), in 2x150 configuration. Sample WNV$_{\text{IRCCS-SCDC_03/2025}}$ produced a total of 11,810,012 reads via the hybrid capture sequencing method (Illumina VSP panel), in 2x150 configuration.

Supplementary Tables

Table S1. Sequencing alignment statistics of the three samples analysed at IRCCS Sacro Cuore Don Calabria. Statistics refer to the alignment against the sequence with GenBank accession ID: PQ654050.

Sample	Enrichment method	Mapping reads	Breath of coverage (%1X)	Breath of coverage (%5X)	Breath of coverage (%10X)	Mean depth of coverage (X)
	VSP panel	394	82	44	13	5

**$WNV_{IRCCS-SCDC\_01/2025}$**	Amplicons	2,193,316	74	67	61	1,300
**$WNV_{IRCCS-SCDC\_02/2025}$**	VSP panel	75,555	100	100	99.95	99.78
**$WNV_{IRCCS-SCDC\_03/2025}$**	VSP panel	18,919	99.95	99.06	98.58	97.11

Supplementary Figure

Figure S1. Complete phylogenetic tree of African-European whole-genomes of WNV-2 (Panel A) and geographical map representing WNV-2a clusters distribution reported in EU countries (Panel B)

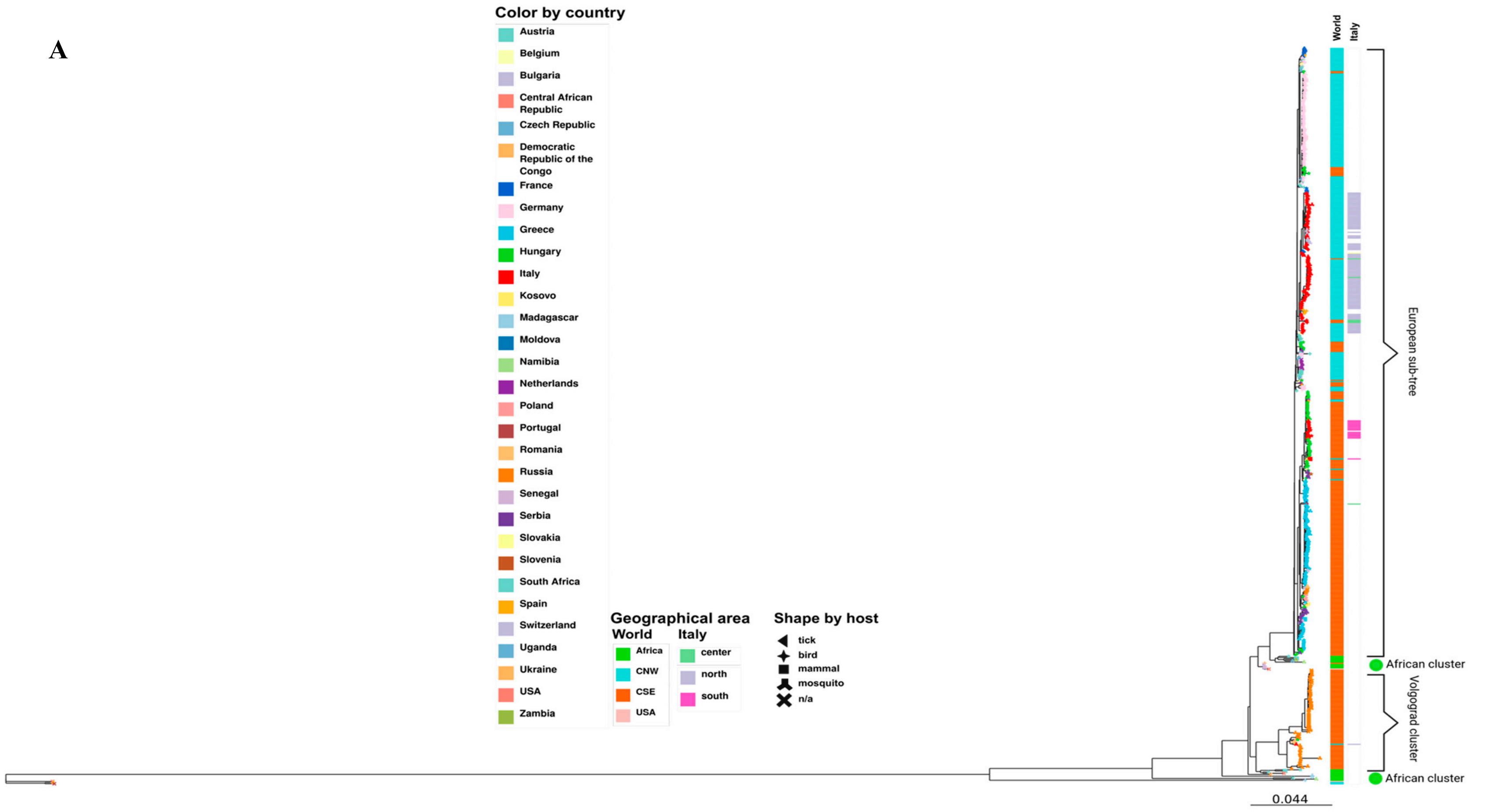

B

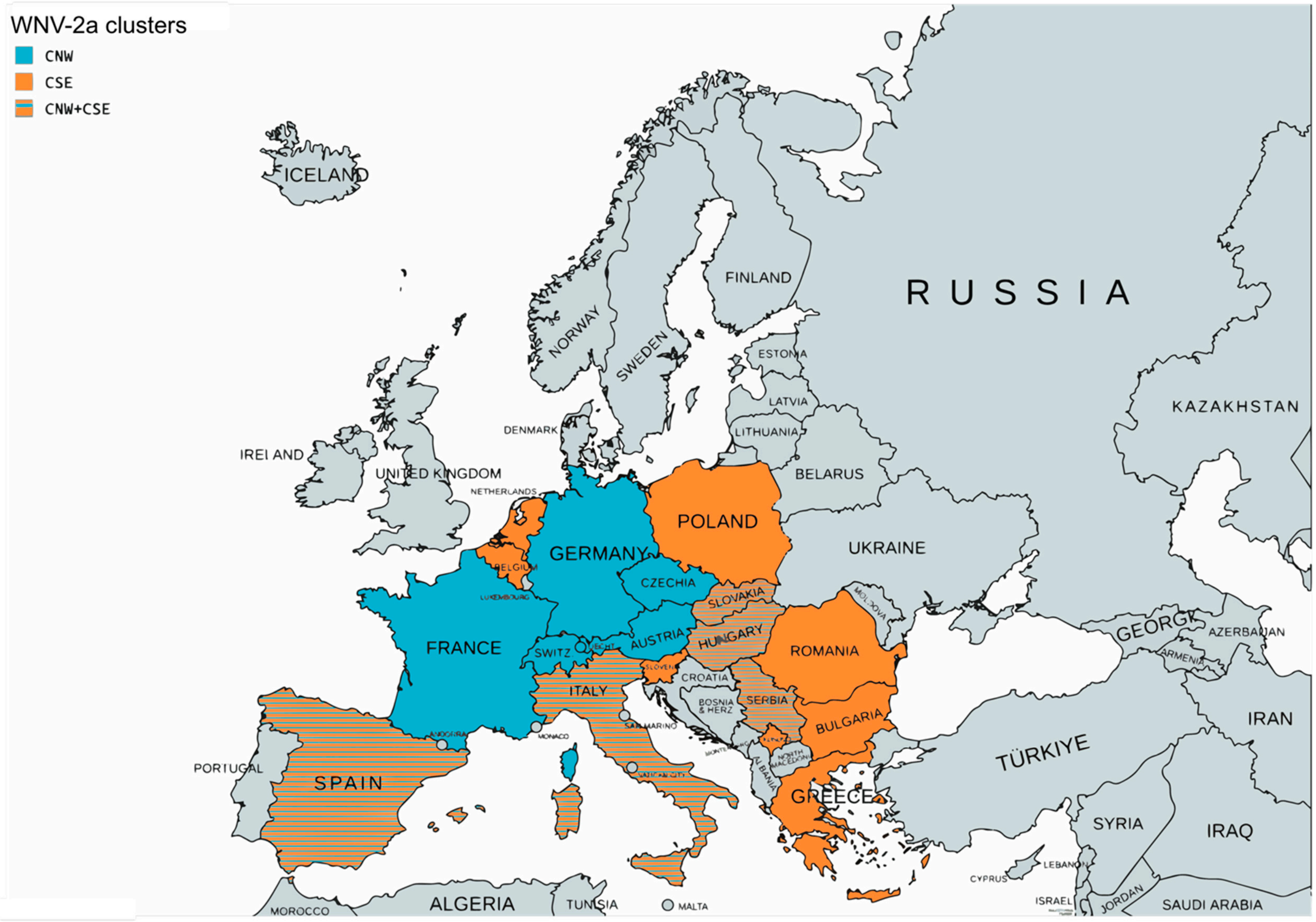

Made with mapchart (https://www.mapchart.net/index.ht
